# 

**Published:** 1893-04-01

**Authors:** 


					THE hospital.
? PRICE TWOPENCE
1 N?v oj/n ?? -
I
-April 1st, 1893.
HM?niiaioiiin t??u?
April* 1b
WITH EXTRA NURSING SUPPLEMEf
Per Annum, 8s. 8d., post free,
Monthly Parts, 6d.; post free, 8d.
Ditto par Annum; 8s., post free.
v WITH fXTRA NUPSIN8 SUPPLEMENT.
No. 340. Vol. XIY.] Saturday, April 1st, 1893.

				

